# Associations between Interleukin Gene Polymorphisms and Risks of Developing Extremity Posttraumatic Osteomyelitis in Chinese Han Population

**DOI:** 10.1155/2020/3278081

**Published:** 2020-05-05

**Authors:** Nan Jiang, Su-yi Li, Yun-fei Ma, Yan-jun Hu, Qing-rong Lin, Bin Yu

**Affiliations:** ^1^Department of Orthopaedics, Nanfang Hospital, Southern Medical University, Guangzhou, China 510515; ^2^Guangdong Provincial Key Laboratory of Bone and Cartilage Regenerative Medicine, Nanfang Hospital, Southern Medical University, Guangzhou, China 510515; ^3^Department of Health Management Center, Nanfang Hospital, Southern Medical University, Guangzhou, China 510515

## Abstract

This case-control study aimed to investigate potential associations between *interleukin* (*IL*) gene polymorphisms and the risks of developing extremity posttraumatic osteomyelitis (PTOM) in Chinese Han population. Altogether, 189 PTOM patients and 200 healthy controls were genotyped of *IL-1α* (rs17561, rs1800587), *IL-1β* (rs16944, rs1143627, rs1143634, rs2853550), *IL-1RN* (rs4251961, rs419598, rs315951), *IL-4* (rs2243248, rs2243250), *IL-6* (rs1800795, rs1800796, rs1800797), *IL-8* (rs4073, rs2227306, rs2227307), *IL-10* (rs3024491, rs3024496, rs1800871, rs1800872, rs1800896), *IL-17A* (rs2275913), and *IL-17F* (rs763780) using the SNaPshot genotyping method. Statistical differences were observed regarding the genotype distributions of rs16944 (*P* = 0.049) and rs4251961 (*P* = 0.007) between the patients and healthy controls. In addition, significant associations were found between rs16944 and the risk of PTOM development by dominant (OR = 1.854, *P* = 0.017), homozygous (OR = 1.831, *P* = 0.041), and heterozygous (OR = 1.869, *P* = 0.022) models, and of rs1143627 by dominant (OR = 1.735, *P* = 0.032) and homozygous (OR = 1.839, *P* = 0.040) models. Moreover, significant links were also identified between rs4251961 and the susceptibility to PTOM by dominant (OR = 0.446, *P* = 0.005) and heterozygous (OR = 0.409, *P* = 0.003) models, and of rs1800796 by dominant (OR = 4.184, *P* = 0.029), homozygous (OR = 4.378, *P* = 0.026), and heterozygous (OR = 3.834, *P* = 0.046) models. The present outcomes demonstrated that rs16944, rs1143627, and rs1800796 associate with increased risks, while rs4251961 links to a decreased risk of PTOM development in Chinese Han population.

## 1. Introduction

Posttraumatic osteomyelitis (PTOM) refers to osseous infection with or without surrounding soft tissue infection following trauma and orthopaedic surgery. It is estimated that the incidence of PTOM ranged between 0.4% and 16.1% [1], with 1% to 2% after closed fractures, and up to 30% following open fractures [2], with the highest rate of 55% [3]. Currently, the accurate diagnosis and successful treatment of PTOM remain challenging in front of clinicians, which primarily attributes to its “wide spectrum” or “high heterogeneity” characteristics. That is to say, clinical presentations may differ among different patients. In addition, clinical efficacy and prognosis are influenced by multiple factors, such as bone location, infection range and duration, pathogen type and virulence, and treatment strategy. The long disease term and a high risk of infection relapse [4] pose patients under great pressure, not only physically and psychologically [5] but also socioeconomically [6]. Therefore, how to lower the incidence is as important as the topic of how to improve the cure rate. To achieve this goal, it is essential to comprehensively understand PTOM pathogenesis, which associates with both environmental and host factors.

Recently, growing evidence has indicated that genetic predisposition acts as an important role in the pathogenesis of PTOM. Wang et al. [7] reported that *cyclooxygenase-2* (*COX-2*) gene polymorphism rs689466 results in an elevated susceptibility to PTOM in Chinese population, with genotype GG as a risk factor. In addition, Alves De Souza et al. [8] indicated that *interleukin* (*IL*) gene polymorphisms rs16944 and rs2234663 may associate with increased risks of developing PTOM in Brazilian. These outcomes imply that single nucleotide polymorphisms (SNPs) involve in PTOM development.

It is known that ILs are a group of cytokines produced and secreted primarily by CD3+ and CD4+ T lymphocytes. They participate in systemic inflammation and play vital roles in innate and adaptive immunity. Previous studies had indicated that *IL* gene SNPs associate with the development of both bacteria- and virus-related inflammatory disorders, such as sepsis [9, 10], periodontitis [11, 12], hepatitis B [13], and C [14] virus infections. However, current studies were still limited regarding the potential associations between *IL* gene SNPs and the risks of developing PTOM, especially in Chinese population. Therefore, this study aimed to assess potential relationships between the twenty-four most frequently detected *IL* gene SNPs and the susceptibilities to PTOM in Chinese Han population.

## 2. Materials and Methods

### 2.1. Patients

We totally included 233 chronic OM patients between August 2013 and October 2015, and 189 of them were as PTOM. PTOM is defined as bone infection with or without surrounding soft tissue infection following trauma and orthopaedic surgery [15]. The diagnosis of PTOM is based on any of the following three confirmatory criteria, histopathological test indicating infection, a definite sinus or fistula connecting directly the bone, or implant, at least two sites of cultures revealing the same pathogen. The control group, with age and sex matched to the patient group, included 147 males and 53 females, with a median age of 41 years, interquartile range (IQR) (36.25, 47). All the participants or their legal guardian signed the informed consent, and this study protocol was approved by medical ethics committee of the hospital.

### 2.2. Measurement of *IL* Gene Polymorphisms

**Figure 1:**
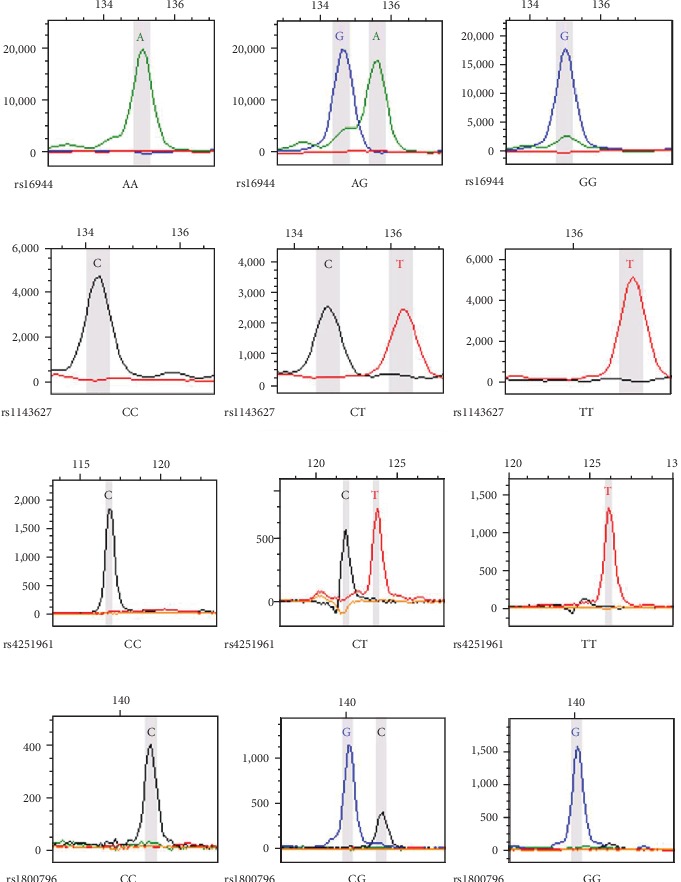
Images of different genotypes of rs16944, 1143627, rs4251961, and rs1800796 using the SNaPshot genotyping method.

Two mL of peripheral blood of each participant was collected in the ethylene diamine tetraacetic acid (EDTA) tube. The genomic DNA was extracted from leukocytes using the salting out method and stored at −80°C. Twenty-four tag SNPs of the *IL* genes were genotyped using the Multiplex SNaPshot system (Applied Biosystems, Foster City, USA). The forward (F), reverse (R), and extension primers used for polymerase chain reactions (PCR) and extension reactions were listed in Table S1. Detail protocols were described previously [16]. The images of different genotypes of rs16944, rs1143627, rs4251961, and rs1800796 using the SNaPshot genotyping method were shown in Figure 1.

### 2.3. Outcome Parameters

Outcome parameters included genotype distribution, mutant allele frequency, and four genetic models (dominant, recessive, homozygous, and heterozygous) of the 24 SNPs in PTOM patients and healthy controls. In addition, the serological levels of IL-6 and tumor necrosis factor *α* (TNF-*α*) before surgery among patients with different genotypes of the SNPs with statistical significances were also reported.

### 2.4. Statistical Analysis

The Statistical Product and Service Solutions software version 13.0 (SPSS Inc., Chicago, IL, USA) was used to conduct the statistical analysis. Data distribution was evaluated for normality by the Kolmogorov–Smirnov test. Continuous variables were presented as mean ± standard deviation (SD) or median with IQR based on data distribution. For normally distributed data, Student's *t* test or one-way analysis of variance (ANOVA) was used to compare between the 2 different groups or among over 2 groups. Otherwise, Mann-Whitney *U* test or Kruskal-Wallis *H* test was applied.

The genotype distributions of the healthy controls were tested for the confirmation to Hardy-Weinberg equilibrium (HWE) using the Chi-square test. The Chi-square test or Fisher exact test was used to compare the genotype distributions and frequencies of mutant allele between the patients and healthy controls. Binary logistic regression analysis with gender, age, and genotype distribution as covariates was used to evaluate potential links between gene polymorphisms and the risks of PTOM development by the four genetic models (dominant, recessive, homozygous, and heterozygous models), with corresponding odds ratios (ORs) and 95% confidence intervals (CIs). All the reported *P* values were 2-sided. A *P* value of less than 0.05 was considered as a statistical significance.

## 3. Results

### 3.1. Clinical Characteristics of the PTOM Patients

Traffic accident (46%) was the most frequent injury type, followed by blunt injury (31%) and a falling injury (12%). Open fractures accounted for two-thirds of the patients, with tibia (51%), femur (30%), and calcaneus (7%) as the top three. The total positive rate of intraoperative specimen cultures was 62%, with *Staphylococcus aureus* (32%) as the most commonly detected pathogen.

### 3.2. HWE Test Outcomes

All the genotyped 24 *IL* gene SNPs were in HWE for healthy controls (*P* > 0.05) (Table S2).

### 3.3. Significant Links between *IL* Genes SNPs and the Susceptibilities to PTOM

#### 3.3.1. *IL-1β* Gene rs16944

Significant difference was found regarding the genotype distribution of rs16944 between the patients and healthy controls (*P* = 0.049). Further comparisons revealed that significant links were established between rs16944 and the risk of developing PTOM by dominant (OR = 1.854, 95% CI 1.118–3.073, *P* = 0.017), homozygous (OR = 1.831, 95% CI 1.026–3.267, *P* = 0.041), and heterozygous (OR = 1.869, 95% CI 1.093–3.194, *P* = 0.022) models, suggesting that population with genotypes of GG and AG may be in a higher risk to develop PTOM (Table 1).

#### 3.3.2. *IL-1β* Gene rs1143627

Significant associations were observed between rs1143627 and the susceptibility to PTOM by dominant (OR = 1.735, 95% CI 1.050–2.868, *P* = 0.032) and homozygous (OR = 1.839, 95% CI 1.029–3.285, *P* = 0.040) models, implying that population with TT genotype may be in an elevated risk of PTOM development (Table 1).

#### 3.3.3. *IL-1RN* Gene rs4251961

Significant difference was identified regarding the genotype distribution of rs4251961 between the patients and controls (*P* = 0.007). The frequency of the mutant allele C in patient group was significantly lower than that in the control group (6.6% vs. 12%, OR = 0.519, 95% CI 0.313–0.816, *P* = 0.01). In addition, significant correlations were found between rs4251961 and the susceptibility to PTOM by dominant (OR = 0.446, 95% CI 0.254–0.781, *P* = 0.005) and heterozygous (OR = 0.409, 95% CI 0.227–0.737, *P* = 0.003) models, indicating that the mutant allele C may be a protective factor against PTOM, and population with CT genotype may have a decreased risk of developing PTOM (Table 1).

#### 3.3.4. *IL-6* Gene rs1800796

Significant relationships was observed between rs1800796 and the risk of PTOM development by dominant (OR = 4.184, 95% CI 1.154–15.165, *P* = 0.029), homozygous (OR = 4.378, 95% CI 1.197–16.007, *P* = 0.026), and heterozygous (OR = 3.834, 95% CI 1.024–14.347, *P* = 0.046) models, demonstrating that population with genotypes of CC and CG may have higher risks of PTOM development (Table 1).

#### 3.3.5. Unestablished Links between Other *IL* Gene SNPs and Susceptibilities to PTOM

Based on this case-control analysis, no significant links were identified between *IL-1α* (rs17561, rs1800587), *IL-1β* (rs1143634, rs2853550), *IL-1RN* (rs419598, rs315951), *IL-4* (rs2243248, rs2243250), *IL-6* (rs1800795, rs1800797), *IL-8* (rs4073, rs2227306, rs2227307), *IL-10* (rs3024491, rs3024496, rs1800871, rs1800872, rs1800896), *IL-17A* (rs2275913), or *IL-17F* (rs763780) and the susceptibilities to PTOM in Chinese Han population (Table S3).

#### 3.3.6. Serological IL-6 and TNF-*α* Levels among Different Genotypes of rs16944, rs1143627, rs4251961, and rs1800796 in the Patient Group

**Figure 2:**
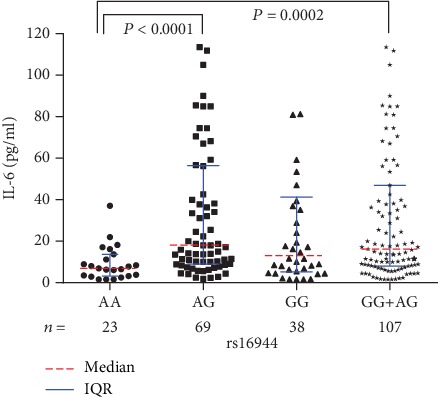
Serological IL-6 levels among different rs16944 genotypes in PTOM patients.

**Figure 3:**
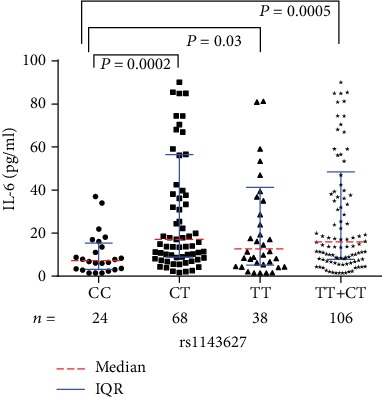
Serological IL-6 levels among different rs1143627 genotypes in PTOM patients.

Significant differences were identified regarding the median serological IL-6 levels among different genotypes of rs16944 (*P* = 0.0006) and rs1143627 (*P* = 0.0014) (Table 2). To be specific, the median IL-6 levels of PTOM patients with AG (*P* < 0.0001) and GG+AG (*P* = 0.0002) genotypes of rs16944 were significantly higher than those with AA genotype (Figure 2). Similarly, the median IL-6 levels of the patients with CT (*P* = 0.0002), TT (*P* = 0.03), and CT+TT (*P* = 0.0005) genotypes of rs1143627 were statistically higher than those with CC genotype (Figure 3).

**Figure 4:**
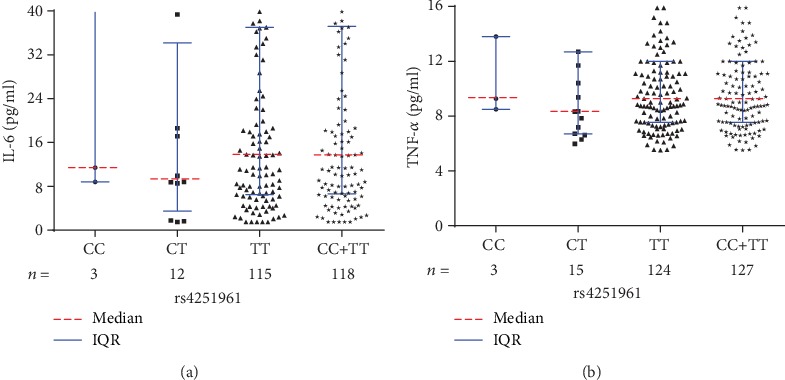
(a) Serological IL-6 levels among different rs4251961 genotypes in PTOM patients. (b) Serological TNF-*α* levels among different rs4251961 genotypes in PTOM patients.

**Figure 5:**
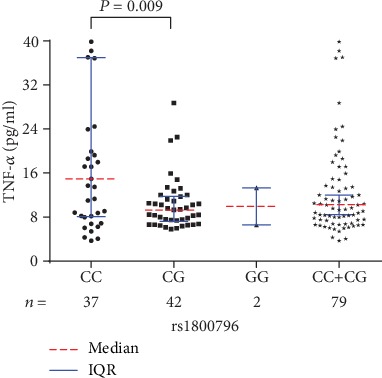
Serological TNF-*α* levels among different rs1800796 genotypes in PTOM patients.

Although no significant difference was found regarding the median serological levels of IL-6 or TNF-*α* among different genotypes of rs4251961, patients with CT genotype had relatively lower median IL-6 and TNF-*α* levels than those with CC and TT genotypes (Figures 4(a) and 4(b)). With respect to rs1800796, significant difference was only identified of the median TNF-*α* level among different genotypes (*P* = 0.030) (Table 2). In addition to a statistically higher median TNF-*α* level in the patients with CC genotype than those with CG genotype (*P* = 0.009) (Figure 5), no significant differences were found regarding the comparisons between another different genotypes, neither for the IL-6 levels nor for the TNF-*α* levels.

## 4. Discussion

The outcomes of the present study demonstrated that, in this Chinese cohort, *IL-1β* gene rs16944 and rs1143627 and *IL-6* gene rs1800796 associate with increased susceptibilities to PTOM. People with genotypes of AG and GG of rs16944, TT of rs1143627, and CG and GG of rs1800796 are in higher risks to develop PTOM. In addition, *IL-1RN* gene rs4251961 is related to a lower risk of PTOM development, with CT genotype as a protective factor. Moreover, patients with genotypes of rs16944 and rs1143627 in higher risks to develop PTOM also had higher serological IL-6 levels.

As mentioned previously, both external and host factors participate in the PTOM pathogenesis. Extrinsic factors, such as injury type and degree, bone location, soft tissue status, pathogen species and virulence, and even early stage treatment strategy, directly affect the incidence of PTOM. In addition to external factors, intrinsic factors also involve in the PTOM development. Most of the previous studies investigated potential roles of host factors from perspective of life styles (e.g., obesity, smoking, and alcohol abuse), immune status (compromised immunity or immune-deficiency), and systematic and local comorbidities (e.g., diabetes, anemia, malignant disorders, venous stasis, and chronic lymphedema) [3, 15, 17–19]. Recently, growing evidence shows that genetic predisposition also participates in the PTOM pathogenesis, with SNPs as an important aspect. Based on the roles of ILs in response to inflammation as well as the already established evidence that *IL* gene SNPs associate with the development of several different inflammatory disorders, we hypothesized that potential links may also exist between *IL* gene SNPs and the PTOM development. Our findings can be summarized with the following four aspects.

First, we found that *IL-1β* gene rs16944 correlates with an increased risk of PTOM development, with AG and GG genotypes as risk factors. The present outcomes share both similarities and differences with previous studies [8, 20, 21]. We agree with the previous conclusions that rs16944 is a risk factor of OM, regardless of OM etiology (trauma, hematogenous spread) and population (Chinese, Saudi, and Brazilian). However, the genotype distributions differed among different investigations, which are probably influenced by OM etiology, ethnicity, and the sample size as well. Although our previous study [20] had reported that rs16944 associates with an increased risk of developing chronic OM, we recruited patients with different causes of chronic OM (infection following trauma and orthopaedic surgery, hematogenous spread, and diabetic foot). Therefore, there may exist a risk of bias. In this study, we only included PTOM patients for analysis. We found, in addition to the AG genotype, GG may be also a risk factor of PTOM. Quite similar to our previous study, the median serological IL-6 levels in PTOM patients with AG and GG+AG genotypes in the present study were also statistically higher than those with AA genotype. This implies that the potential mechanisms of rs16944 involving in PTOM may be partially via the regulation of IL-6 level.

Second, we found that *IL-1β* gene rs1143627 also associates with an elevated susceptibility to PTOM, with TT genotype as a risk factor. While in the previous study [20], we failed to obtain any significant links between the two, which is probably because of the inclusion of the non-PTOM patients. Although no statistical differences were identified regarding the genotype distribution, mutant allele frequency, or the heterozygous model between the two groups, their *P* values were close to 0.05, implying that the mutant allele T may be a risk factor and population with CT genotype may be in a higher risk to develop PTOM. However, studies with a larger sample size are warranted to achieve more accurate conclusions. Similar to rs16944, the median IL-6 levels in patients with CT, TT, and CT+TT genotypes of rs1143627 were significantly higher than those with CC genotype, suggesting a potentially similar mechanism to that of rs16944.

Third, we found that *IL-1RN* gene rs4251961 is linked to a decreased risk of developing PTOM, with CT genotype as a protective factor. It is known that *IL-1RN* encodes the IL-1 receptor antagonist (IL-1ra), an endogenous immunomodulatory cytokine that inhibits the actions of IL-1*α* and IL-1*β*. *IL-1RN* gene polymorphisms affect IL-1ra level and thus may influence the risk of infection. Similar to our study, Beck et al. [22] reported that the minor C allele of rs4251961 was independently associated with a decreased risk of infection (apart from pneumonia) after stroke. Here, the serological IL-1ra levels were not detected, and therefore, whether significant differences exist regarding the IL-ra levels among different rs4251961 genotypes remain unclear. However, we compared serum IL-6 and TNF-*α* levels among different genotypes of rs4251961. Although no statistical differences were obtained, patients with CT genotype had relatively lower levels of IL-6 and TNF-*α* than those with CC and TT genotypes. Potential mechanisms should be further explored.

Fourth, we found that *IL-6* gene rs1800796 is related to an increased susceptibility to PTOM, with genotypes of CC and CG as risk factors. Similar to rs1143627, although no statistical differences were found of the genotype distribution or mutant allele frequency between the two groups, their *P* values were close to 0.05, demonstrating the mutant allele C is probably a risk factor of PTOM. Besides, no statistical difference was found of the IL-6 level among different genotypes, which may be primarily because of the too limited sample size of the GG genotype (3 cases). With regard to the TNF-*α* level, significant difference was identified among different genotypes of rs1800796 in PTOM patients. The outcomes of the posthoc multiple comparisons revealed that only patients with CC genotype had a significantly higher TNF-*α* level than those with CG genotype. Considering the limited sample size of GG genotype in this study, cautious attitude should be taken towards the results.

Previous studies [23, 24] had reported that *IL-1α* (rs1800587), *IL-4* (rs2243248, rs2243250), and *IL-6* (rs1800795) SNPs associate with increased susceptibilities to OM. However, our study did not find any significant links between the four SNPs and the risks of PTOM development, which may be associated with different ethnicities among different studies. In addition, we also failed to find any significant links between *IL-1α* (rs17561), *IL-1β* (rs1143634, rs2853550), *IL-1RN* (rs419598, rs315951), *IL-6* (rs1800797), *IL-8* (rs4073, rs2227306, rs2227307), *IL-10* (rs3024491, rs3024496, rs1800871, rs1800872, rs1800896), *IL-17A* (rs2275913), or I*L-17F* (rs763780) and the risks of developing PTOM in Chinese population.

In this Chinese cohort, all of the four SNPs with significant links to the development of PTOM locate in their promoter regions, and thus, they may alter expression levels of the respective encoding products, the latter of which may participate in the pathogenesis of PTOM. In addition to being related to the PTOM development, such SNPs may also influence clinical efficacy of the PTOM treatment. It is known that the infection recurrence rates following bone infection have been reported to be as high as 20% to 30% [25, 26]; therefore, whether such SNPs also affect the risk of infection recurrence requires future investigations. As the current study revealed that four *IL* gene SNPs may involve in the PTOM pathogenesis, one potential application is using such SNPs for evaluating the risk of PTOM development in advance in some patients. However, we believe that before the implementation of this application, at least two essential steps should be performed. The first step is enlarging the sample size to obtain more confirmatory relationships between such SNPs and the risks of PTOM development. The following step is conducting a cohort study to comprehensively assess the predictive values of such SNPs for PTOM.

The present study also had several limitations. First, the sample size remains limited as a genome-wide association study (GWAS). In order to obtain more reliable outcomes, future studies with a larger sample size are necessary. Second, we did not detect serological levels of IL-1*β* or IL-1ra, and therefore, relationships between different genotypes of rs16944, rs1143627, and IL-1*β* levels, as well as associations between the different genotypes of rs4251961 and IL-1ra levels, remain unclear. Last, this study was only a preliminary report; the detailed mechanisms of the four SNPs in the pathogenesis of PTOM should be further investigated.

## 5. Conclusion

In summary, in this Chinese cohort, *IL-1β* gene rs16944, rs1143627, and *IL-6* gene rs1800796 associate with increased risks of developing PTOM, with genotypes of GG and AG of rs16944, TT of rs1143627, and CC and CG of rs1800796 as risk factors. However, *IL-RN* gene rs4251961 is related to a decreased susceptibility to PTOM, with genotype of CT as a protective factor.

## Figures and Tables

**Table 1:** Comparisons of genotype distribution, allele frequency, and genetic models of rs16944, rs1143627, rs4251961, and rs1800796 between PTOM patients and healthy controls.

SNP	Item		Patients	Controls	Test statistics	*P* values	OR (95% CI)
rs16944	Genotype (*n*, %)	AA	30 (15.87)	52 (26.0)	6.012	**0.049**	
		AG	98 (51.85)	90 (45.0)			
		GG	61 (32.28)	58 (29.0)			
	Allele frequency	G vs. A	220/158	206/194	3.523	0.061	1.311 (0.988-1.741)
	Dominant model	GG+AG vs. AA	159/30	148/52	5.733	**0.017**	1.854 (1.118-3.073)
	Recessive model	GG vs. AA+AG	61/128	58/142	0.555	0.456	1.180 (0.764-1.822)
	Homozygous model	GG vs. AA	61/30	58/52	4.184	**0.041**	1.831 (1.026-3.267)
	Heterozygous model	AG vs. AA	98/30	90/52	5.225	**0.022**	1.869 (1.093-3.194)

rs1143627	Genotype (*n*, %)	CC	31 (16.40)	51 (25.5)	4.928	0.085	
		CT	97 (51.32)	94 (47.0)			
		TT	61 (32.28)	55 (27.5)			
	Allele frequency	T vs. C	219/159	204/196	3.769	0.052	1.323 (0.997-1.756)
	Dominant model	TT+CT vs. CC	158/31	149/51	4.615	**0.032**	1.735 (1.050-2.868)
	Recessive model	TT vs. CC + CT	61/128	55/145	1.202	0.273	1.278 (0.824-1.981)
	Homozygous model	TT vs. CC	61/31	55/51	4.229	**0.040**	1.839 (1.029-3.285)
	Heterozygous model	CT vs. CC	97/31	94/51	3.606	0.058	1.675 (0.984-2.853)

rs4251961	Genotype (*n*, %)	TT	167 (88.36)	155 (77.5)	8.967	**0.007**	
		CT	19 (10.05)	42 (21.0)			
		CC	3 (1.59)	3 (1.5)			
	Allele frequency	C vs. T	25/353	48/352	6.631	**0.010**	0.519 (0.313-0.861)
	Dominant model	CC+CT vs. TT	22/167	45/155	7.980	**0.005**	0.446 (0.254-0.781)
	Recessive model	CC vs. TT+CT	3/186	3/197	0.038	0.845	1.177 (0.231-6.007)
	Homozygous model	CC vs. TT	3/167	3/155	0.000	0.985	1.016 (0.198-5.214)
	Heterozygous model	CT vs. TT	19/167	42/155	8.844	**0.003**	0.409 (0.227-0.737)

rs1800796	Genotype (*n*, %)	GG	3 (1.59)	12 (6.0)			
		CG	58 (30.69)	64 (32.0)	5.452	0.065	
		CC	128 (67.72)	124 (62.0)			
	Allele frequency	C vs. G	314/64	312/88	3.176	0.075	1.384 (0.967-1.980)
	Dominant model	CC+CG vs. GG	186/3	188/12	4.745	**0.029**	4.184 (1.154-15.165)
	Recessive model	CC vs. GG+CG	128/61	124/76	1.433	0.231	1.295 (0.848-1.979)
	Homozygous model	CC vs. GG	128/3	124/12	4.983	**0.026**	4.378 (1.197-16.007)
	Heterozygous model	CG vs. GG	58/3	64/12	3.984	**0.046**	3.834 (1.024-14.347)

PTOM: posttraumatic osteomyelitis; OR: odds ratio; CI: confidence interval.

**Table 2:** Serological levels of IL-6 and TNF-*α* among different genotypes of rs16944, rs1143627, rs4251961, and rs1800796 in patient group.

Items	rs16944				rs1143627				rs4251961				rs1800796			
	AA	AG	GG	*P* value	CC	CT	TT	*P* value	TT	CT	CC	*P* value	GG	CG	CC	*P* value
IL-6 (pg/ml) median (IQR)	6.87 (3.16, 13.6)	17.92 (8.92, 56.41)	13.04 (5.19, 41.25)	**0.0006**	7.32 (3.24, 15.49)	17.56 (8.84, 56.53)	13.04 (5.19, 41.25)	**0.0014**	13.8 (6.49, 37.02)	9.37 (3.48, 34.17)	11.4 (8.8, 151.4)	0.666	72.07 (2.73, 141.4)	14.96 (8.08, 36.94)	13.6 (6.19, 37.73)	0.805
TNF-*α* (pg/ml) median (IQR)	9.67 (7.85, 10.9)	9.78 (7.69, 12.75)	5 (3.15, 74)	0.724	9.67 (8.04, 11.05)	9.60 (7.63, 12.88)	8.50 (7.2, 12.35)	0.676	9.29 (7.55, 12)	8.35 (6.71, 12.7)	9.28 (8.5, 13.8)	0.795	9.94 (6.58, 13.3)	9.37 (7.23, 11.78)	14.96 (8.08, 36.94)	**0.030**

IQR: interquartile range.

## Data Availability

The datasets generated and/or analyzed during the current study are not publicly available due to the respect and protection of privacy of the patients, but are available from the corresponding author on reasonable request.

## Supplementary Materials

Supplementary Materials Table S1: PCR primers and extension primers of the *interleukin* gene polymorphisms. Table S2: HWE test outcomes of the *IL* gene polymorphisms for healthy controls. Table S3: comparisons of genotype distribution, allele frequency, and genetic models of the remaining 20 SNPs between PTOM patients and healthy controls.
