# Supplementary material for: Associations between Interleukin Gene Polymorphisms and Risks of Developing Extremity Posttraumatic Osteomyelitis in Chinese Han Population
Source: Mediators Inflamm. 2020 May 5;2020:3278081. doi: 10.1155/2020/3278081 (PMC7222541; doi:10.1155/2020/3278081)
Supplement: Supplementary Materials — Table S1: PCR primers and extension primers of the interleukin gene polymorphisms. Table S2: HWE test outcomes of the IL gene polymorphisms for healthy controls. Table S3: comparisons of genotype distribution, allele frequency, and genetic models of the remaining 20 SNPs between PTOM patients and healthy controls. [file 3278081.f1.pdf]

Table S1 PCR primers and extension primers of the *IL* gene polymorphisms

SNP	PCR primers	Extension primers
rs16944	F: 5’-ATGTGGGACAAAGTGGAAGAC-3’	5’-TTTTTTTTTTTTCTTGGGTGCTGTTCTCTGCCTC-3’
	R: 5’-CAATTTTCTCCTCAGAGGCTC-3’	
rs1143627	F: 5’-TCCACCAATACTCTTTTCCCC-3’	5’-TTTTTTTTTTTTTTTTTCCTACTTCTGCTTTTGAAAGC-3’
	R: 5’-CCCTTAGCACCTAGTTGTAAG-3’	
rs1143634	F: 5’-CTACTGGTGTTGTCATCAGAC-3’	5’-TTTTTTTTTTTTTTTTTTAAGCCTCGTTATCCCATGTGTC-3’
	R: 5’-AGCTTTTTTGCTGTGAGTCCC-3’	
rs2853550	F: 5’-ACTAAAGCCCACTCCTCATTG-3’	5’-TTTTTTTTTTTTTTTTTTTTTCAGAAGGATATTCAGTGCACAT-3’
	R: 5’-ATAGCGCTTGCTCAACAGATG-3’	
rs1800587	F: 5’-GTGTTCTACCACCTGAACTAG-3’	5’-TTTTTTTTTTTTTTTTTTTTTTTTTTTACATATGAGCCTTCAATG-3’
	R: 5’-ATATGCCCAAGGTGTGTCTTC-3’	
rs17561	F: 5’-CCTCCAGAACTATTTTCCCTG-3’	5’-TTTTTTTTTTTTTTTTAGAAATCATCAAGCCTAGGTCA-3’
	R: 5’-TGAGGTACTGATCATTGGCTC-3’	
rs4251961	F: 5’-GATTGTAGGTGGCAAGATTGG-3’	5’-GTCTAAGATAGGGCAGATAGCA-3’
	R: 5’-TGCCCTTCAGACCTCATTTTG-3’	
rs419598	F: 5’-CACTACAGCTGAGTCCTTTTC-3’	5’-CATTTGGTCCTTGCAAGTATCC-3’
	R: 5’-CAAGGATTAGGACATTGCACC-3’	
rs315951	F: 5’-TAGAAAGACCATTCTGGAGGC-3’	5’-TTTTTTTTGGCAGAGTCCTGTGACCAGGTT-3’
	R: 5’-AATTCTACTTCCAGGAGGACG-3’	
rs2243248	F: 5’-ACTGACTAGGAGGGCTGATTTG-3’	5’-TTTTTTTTTTTTTTTTCTGATTTGTAAGTTGGTAAGAC-3’
	R: 5’-TATGTTGCCTAGGCTCATCTC-3’	
rs2243250	F: 5’-CCTCAGAATAGACCTACCTTG-3’	5’-CCTAAACTTGGGAGAACATTGT-3’
	R: 5’-CATCTTGGAAACTGTCCTGTC-3’	
rs1800795	F: 5’-CGCTAGCCTCAATGACGACCT-3’	5’-TTTTTTTTGCAATGTGACGTCCTTTAGCAT-3’

	R: 5’-TTGAGACTCTAATATTGAGAC-3’	
rs1800796	F: 5’-AGGTGAAGAAAGTGGCAGAAG-3’	5’-TTTTTTTTTTTTTTTTTTTTGTGTTCTGGCTCTCCCTGTGAG-3’
	R: 5’-AAGAAGGTAATACTACCAGTC-3’	
rs1800797	F: 5’-AGGTGAAGAAAGTGGCAGAAG-3’	5’-CTGTTGTAGAACTGCCTGGCCA-3’
	R: 5’-AAGAAGGTAATACTACCAGTC-3’	
rs4073	F: 5’-GATCTTGTTCTAACACCTGCC-3’	5’-TTTTTTTTTTCCACAATTTGGTGAATTATCAA-3’
	R: 5’-AACCTGAGTCATCACACTTCC-3’	
rs2227306	F: 5’-GGCAATTTCTATGCTGGAGAG-3’	5’-GTCATAACTGACAACATTGAAC-3’
	R: 5’-TCCTGAATATTCTCCTAGCCC-3’	
rs2227307	F: 5’-GATGCTTTGGTAACAAACATCC-3’	5’-TTTTTTTTTTTTTTTTTTTACGTTAAATATATGCATGCTAC-3’
	R: 5’-GGTAACCGTGGTTCTCAATAG-3’	
rs3024491	F: 5’-TTTCCAGACCTGAAAGACCAG-3’	5’-TTTTTTTTTTTTTTTTTGACATAGGTGTCCCTTAAAGCC-3’
	R: 5’-ACTCAGTCCTGGTCTTCTTTC-3’	
rs3024496	F: 5’-AGAGGTCTCCAAAATCGGATC-3’	5’-AGAAACCTTATTGTACCTCTCT-3’
	R: 5’-TGTGTCACCCTATGGAAACAG-3’	
rs1800871	F: 5’-AACTTTAGACTCCAGCCACAG-3’	5’-GAGCAAACTGAGGCACAGAGAT-3’
	R: 5’-TATGCTAGTCAGGTAGTGCTC-3’	
rs1800872	F: 5’-AGAGGTGGAAACATGTGCCTG-3’	5’-TTTTTTTTTTTTTTTCACATCCTGTGACCCCGCCTGT-3’
	R: 5’-CTTGCTAACTTAGGCAGTCAC-3’	
rs1800896	F: 5’-ATCCAAGACAACACTACTAAG-3’	5’-CACTACTAAGGCTTCTTTGGGA-3’
	R: 5’-TGTAAGCTTCTGTGGCTGGAG-3’	
rs2275913	F: 5’-CTCTGCTCAGCTTCTAACAAG-3’	5’-TTTTTTTTTTCCTTCCCATTTTCCTTCAGAAG-3’
	R: 5’-GGACAAAATGTAGCGCTATCG-3’	
rs763780	F: 5’- TTTCTGTTTCCATCCGTGCAG-3’	5’-TTTTTTTTGATATGCACCTCTTACTGCACA-3’
	R: 5’-CAAGGCTGCTCTGTTTCTTTC-3’	

IL: Interleukin ; SNP: Single Nucleotide Polymorphism; PCR: Polymerase Chain Reaction; F: Forward; R: Reverse.

Table S2 HWE test outcomes of the *IL* gene polymorphisms for healthy controls

**SNP**	**χ^2 value**	***P* value**
rs16944	1.968	0.161
rs1143627	0.711	0.399
rs1143634	0.046	0.829
rs2853550	0.624	0.429
rs1800587	0.497	0.481
rs17561	0.497	0.481
rs4251961	0.006	0.936
rs419598	0.744	0.388
rs315951	0.833	0.361
rs2243248	0.221	0.638
rs2243250	1.641	0.200
rs1800795	0.000	1.000
rs1800796	0.914	0.339
rs1800797	0.001	0.972
rs4073	0.344	0.557
rs2227306	1.183	0.277
rs2227307	0.584	0.445
rs3024491	1.232	0.267
rs3024496	0.958	0.327
rs1800871	0.002	0.963
rs1800872	0.080	0.778
rs1800896	0.995	0.318
rs2275913	0.134	0.714
rs763780	1.423	0.233

IL: Interleukin; HWE: Hardy-Weinber Equilibrium

Table S3 Comparisons of genotype distribution, allele frequency, and genetic models of the remaining 20 SNPs between PTOM patients and healthy controls

SNP	Item		Patients	Controls	Test statistics	*P* values	OR (95% CI)
rs1143634	Genotype (n, %)	CC	185 (97.88)	194 (97.0)			
		CT	4 (2.12)	6 (3.0)	NA	0.752	
		TT	0 (0.0)	0 (0.0)			
	Allele frequency	T vs. C	4/374	6/394	0.052	0.819	0.702 (0.197-2.508)
	Dominant model	TT+CT vs. CC	4/185	6/194	0.240	0.624	0.725 (0.200-2.629)
	Recessive model	TT vs. CC+CT	0/189	0/200	NA	NA	NA
	Homozygous model	TT vs. CC	0/185	0/194	NA	NA	NA
	Heterzygous model	CT vs. CC	4/185	6/194	0.240	0.624	0.725 (0.200-2.629)
rs2853550	Genotype (n, %)	CC	168 (88.89)	171 (85.5)			
		CT	21 (11.11)	27 (13.5)	2.093	0.352	
		TT	0 (0.0)	2 (1.00)			
	Allele frequency	T vs. C	21/357	31/369	1.501	0.221	0.700 (0.395-1.242)
	Dominant model	TT+CT vs. CC	21/168	29/171	1.232	0.267	0.710 (0.388-1.300)
	Recessive model	TT vs. CC+CT	0/199	2/198	0.000	0.999	0.000 (0.000-
	Homozygous model	TT vs. CC	0/168	2/171	0.000	0.999	0.000 (0.000-
	Heterzygous model	CT vs. CC	21/168	27/171	0.730	0.393	0.766 (0.415-1.413)
rs1800587	Genotype (n, %)	CC	171 (90.48)	181 (90.5)			
		CT	16 (8.46)	19 (9.5)	1.867	0.467	
		TT	2 (1.06)	0 (0.0)			
	Allele frequency	T vs. C	20/358	19/381	0.119	0.730	1.120 (0.588-2.134)

	Dominant model	TT+CT vs. CC	18/171	19/181	0.005	0.942	0.975 (0.493-1.929)
	Recessive model	TT vs. CC+CT	2/187	0/200	0.000	0.999	/
	Homozygous model	TT vs. CC	2/171	0/181	0.000	0.999	/
	Heterzygous model	CT vs. CC	16/171	19/181	0.189	0.664	0.856 (0.424-1.728)
rs17561	Genotype (n, %)	GG	171 (90.48)	181 (90.5)			
		GT	17 (8.99)	19 (9.5)	1.031	0.860	
		TT	1 (0.53)	0 (0.0)			
	Allele frequency	T vs. G	19/359	19/381	0.032	0.858	1.061 (0.553-2.037)
	Dominant model	TT+GT vs. GG	18/171	19/181	0.001	0.980	0.991 (0.501-1.961)
	Recessive model	TT vs. GG+GT	1/181	0/200	0.000	1.000	/
	Homozygous model	TT vs. GG	1/171	0/181	0.000	1.000	/
	Heterzygous model	GT vs. GG	17/171	19/181	0.051	0.821	0.923 (0.462-1.844)
rs419598	Genotype (n, %)	TT	158 (83.60)	177 (88.5)			
		CT	30 (15.87)	23 (11.5)	2.620	0.210	
		CC	1 (0.53)	0 (0.0)			
	Allele frequency	C vs. T	32/346	23/377	2.182	0.140	1.516 (0.870-2.642)
	Dominant model	CC+CT vs. TT	31/158	23/177	1.442	0.230	1.431 (0.797-2.569)
	Recessive model	CC vs. TT+CT	1/188	0/200	0.000	1.000	/
	Homozygous model	CC vs. TT	1/158	0/177	0.000	1.000	/
	Heterzygous model	CT vs. TT	30/158	23/177	1.203	0.273	1.390 (0.772-2.504)
rs315951	Genotype (n, %)	GG	35 (18.52)	38 (19.0)			
		CG	91 (48.15)	91 (45.5)	0.290	0.865	
		CC	63 (33.33)	71 (35.5)			

	Allele frequency	C vs. G	217/161	233/167	0.057	0.812	0.966 (0.727-1.284)
	Dominant model	CC+CG vs. GG	154/35	162/38	0.001	0.982	1.006 (0.602-1.681)
	Recessive model	CC vs. GG+CG	63/126	71/129	0.153	0.696	0.919 (0.603-1.402)
	Homozygous model	CC vs. GG	63/35	71/38	0.029	0.864	0.951 (0.535-1.690)
	Heterzygous model	CG vs. GG	91/35	91/38	0.029	0.865	1.049 (0.606-1.814)
rs2243248	Genotype (n, %)	TT	157 (83.07)	166 (83.0)			
		GT	31 (16.40)	33 (16.5)	0.253	1.000	
		GG	1 (0.53)	1 (0.5)			
	Allele frequency	G vs. T	33/345	35/365	0.000	0.992	0.998 (0.606-1.641)
	Dominant model	GG+GT vs. TT	32/157	34/166	0.026	0.872	1.045 (0.612-1.786)
	Recessive model	GG vs. TT+GT	1/188	1/199	0.008	0.931	1.133 (0.068-18.841)
	Homozygous model	GG vs. TT	1/157	1/166	0.009	0.926	1.142 (0.068-19.032)
	Heterzygous model	GT vs. TT	31/157	33/166	0.022	0.881	1.042 (0.606-1.793)
rs2243250	Genotype (n, %)	CC	5 (2.65)	6 (3.0)			
		CT	55 (29.10)	73 (36.5)	2.569	0.277	
		TT	129 (68.25)	121 (60.5)			
	Allele frequency	T vs. C	313/65	315/85	2.053	0.152	1.299 (0.908-1.860)
	Dominant model	TT+CT vs. CC	184/5	194/6	0.002	0.967	1.026 (0.302-3.481)
	Recessive model	TT vs. CC+CT	129/60	121/79	2.119	0.146	1.366 (0.897-2.081)
	Homozygous model	TT vs. CC	129/5	121/6	0.049	0.826	1.148 (0.336-3.929)
	Heterzygous model	CT vs. CC	55/5	73/6	0.087	0.768	0.828 (0.236-2.903)
rs1800795	Genotype (n, %)	GG	187 (99.47)	200 (100)			
		CG	1 (0.53)	0 (0.0)	NA	0.485	

		CC	0 (0.0)	0 (0.0)			
	Allele frequency	C vs. G	1/375	0/400	NA	0.485	0.997 (0.992-1.003)
	Dominant model	CC+CG vs. GG	1/187	0/200	0.000	1.000	/
	Recessive model	CC vs. GG+CG	0/188	0/200	NA	NA	NA
	Homozygous model	CC vs. GG	0/187	0/200	NA	NA	NA
	Heterzygous model	CG vs. GG	1/187	0/200	0.000	1.000	/
rs1800797	Genotype (n, %)	GG	187 (98.94)	199 (99.5)			
		AG	2 (1.06)	1 (0.5)	NA	0.614	
		AA	0 (0.0)	0 (0.0)			
	Allele frequency	A vs. G	2/376	1/399	0.002	0.961	2.122 (0.192-23.502)
	Dominant model	AA+AG vs. GG	2/187	1/199	0.257	0.612	1.866 (0.167-20.823)
	Recessive model	AA vs. GG+AG	0/189	0/200	NA	NA	NA
	Homozygous model	AA vs. GG	0/187	0/199	NA	NA	NA
	Heterzygous model	AG vs. GG	2/187	1/199	0.257	0.612	1.866 (0.167-20.823)
rs4073	Genotype (n, %)	AA	37 (19.58)	32 (16.0)			
		AT	77 (40.74)	91 (45.5)	1.245	0.537	
		TT	75 (39.68)	77 (38.5)			
	Allele frequency	T vs. A	227/151	245/155	0.117	0.733	0.951 (0.713-1.268)
	Dominant model	TT+AT vs. AA	152/37	168/32	1.190	0.275	0.745 (0.439-1.265)
	Recessive model	TT vs. AA+AT	75/114	77/123	0.004	0.952	1.013 (0.671-1.529)
	Homozygous model	TT vs. AA	75/37	77/32	0.633	0.426	0.790 (0.443-1.411)
	Heterzygous model	AT vs. AA	77/37	91/32	1.429	0.232	0.707 (0.400-1.248)
rs2227306	Genotype (n, %)	CC	94 (49.74)	81 (40.5)			

		CT	74 (39.15)	98 (49.0)	4.007	0.135	
		TT	21 (11.11)	21 (10.5)			
	Allele frequency	T vs. C	116/262	140/260	1.637	0.201	0.822 (0.609-1.110)
	Dominant model	TT+CT vs. CC	95/94	119/81	2.609	0.106	0.716 (0.477-1.074)
	Recessive model	TT vs. CC+CT	21/168	21/179	0.080	0.778	1.098 (0.575-2.097)
	Homozygous model	TT vs. CC	21/94	21/81	0.088	0.767	0.902 (0.456-1.784)
	Heterzygous model	CT vs. CC	78/94	98/81	3.206	0.073	0.677 (0.441-1.038)
rs2227307	Genotype (n, %)	TT	76 (40.21)	74 (37.0)			
		GT	78 (41.27)	91 (45.5)	0.716	0.699	
		GG	35 (18.52)	35 (17.5)			
	Allele frequency	G vs. T	148/230	161/239	0.098	0.755	0.955 (0.717-1.273)
	Dominant model	GG+GT vs. TT	113/76	126/74	0.202	0.653	0.909 (0.601-1.376)
	Recessive model	GG vs. TT+GT	35/154	35/165		0.648	1.130 (0.668-1.912)
	Homozygous model	GG vs. TT	35/76	35/74	0.020	0.887	1.043 (0.585-1.858)
	Heterzygous model	GT vs. TT	78/76	91/74	0.435	0.509	0.861 (0.552-1.343)
rs3024491	Genotype (n, %)	GG	178 (94.18)	184 (92.0)			
		GT	11 (5.82)	15 (7.5)	1.339	0.548	
		TT	0 (0.0)	1 (0.5)			
	Allele frequency	T vs. G	11/367	17/383	1.006	0.316	0.675 (0.312-1.461)
	Dominant model	TT+GT vs. GG	11/178	16/184	0.271	0.603	0.806 (0.357-1.816)
	Recessive model	TT vs. GG+GT	0/189	1/199	NA	NA	NA
	Homozygous model	TT vs. GG	0/178	1/184	NA	NA	NA
	Heterzygous model	GT vs. GG	11/178	15/184	0.271	0.603	0.806 (0.357-1.816)

rs3024496	Genotype (n, %)	CC	0 (0.0)	1 (0.5)			
		CT	11 (5.82)	16 (8.0)	1.611	0.431	
		TT	178 (94.18)	183 (91.5)			
	Allele frequency	T vs. C	367/11	382/18	1.369	0.242	1.572 (0.733-3.374)
	Dominant model	TT+CT vs. CC	189/0	199/1	0.000	1.000	/
	Recessive model	TT vs. CC+CT	178/11	183/17	0.788	0.375	1.433 (0.648-3.169)
	Homozygous model	TT vs. CC	178/0	183/1	0.000	1.000	/
	Heterzygous model	CT vs. CC	11/0	16/1	0.000	1.000	/
rs1800871	Genotype (n, %)	CC	15 (7.94)	25 (12.5)			
		CT	89 (47.09)	91 (45.5)	2.219	0.330	
		TT	85 (44.97)	84 (42.0)			
	Allele frequency	T vs. C	259/119	259/141	1.240	0.265	1.185 (0.879-1.597)
	Dominant model	TT+CT vs. CC	174/15	175/25	2.370	0.124	1.704 (0.865-3.357)
	Recessive model	TT vs. CC+CT	85/104	84/116	0.204	0.651	1.098 (0.733-1.645)
	Homozygous model	TT vs. CC	85/15	84/25	2.138	0.144	1.700 (0.835-3.464)
	Heterzygous model	CT vs. CC	89/15	91/25	2.180	0.140	1.707 (0.839-3.470)
rs1800872	Genotype (n, %)	CC	15 (7.94)	25 (12.5)			
		AC	88 (46.56)	94 (47.0)	2.538	0.281	
		AA	86 (45.50)	81 (40.5)			
	Allele frequency	A vs. C	260/118	256/144	1.991	0.158	1.239 (0.920-1.670)
	Dominant model	AA+AC vs. CC	174/15	175/25	2.370	0.124	1.704 (0.865-3.357)
	Recessive model	AA vs. CC+AC	86/103	81/119	0.699	0.403	1.189 (0.793-1.783)
	Homozygous model	AA vs. CC	86/15	84/25	2.513	0.113	1.779 (0.873-3.628)

	Heterzygous model	AC vs. CC	88/15	94/25	1.848	0.174	1.635 (0.805-3.322)
rs1800896	Genotype (n, %)	AA	165 (87.30)	173 (86.5)			
		AG	24 (12.70)	25 (12.5)	1.538	0.642	
		GG	0 (0.0)	2 (1.0)			
	Allele frequency	G vs. A	24/354	29/371	0.248	0.618	0.867 (0.495-1.519)
	Dominant model	GG+AG vs. AA	24/165	27/173	0.012	0.912	0.967 (0.532-1.756)
	Recessive model	GG vs. AA+AG	0/189	2/198	0.000	0.999	/
	Homozygous model	GG vs. AA	0/165	2/173	0.000	0.999	/
	Heterzygous model	AG vs. AA	24/165	25/173	0.030	0.862	1.055 (0.575-1.937)
rs2275913	Genotype (n, %)	AA	43 (22.75)	45 (22.5)			
		AG	104 (55.03)	97 (48.5)	2.540	0.281	
		GG	42 (22.22)	58 (29.0)			
	Allele frequency	G vs. A	188/190	213/187	0.961	0.327	0.869 (0.656-1.151)
	Dominant model	GG+AG vs. AA	146/43	155/45	0.001	0.978	1.007 (0.624-1.625)
	Recessive model	GG vs. AA+AG	42/147	58/142	3.024	0.082	0.662 (0.416-1.054)
	Homozygous model	GG vs. AA	42/43	58/45	1.027	0.311	0.741 (0.414-1.324)
	Heterzygous model	AG vs. AA	104/43	97/45	0.395	0.530	1.177 (0.708-1.954)
rs763780	Genotype (n, %)	TT	132 (69.84)	145 (72.5)			
		CT	51 (26.98)	53 (26.5)	2.238	0.326	
		CC	6 (3.17)	2 (1.0)			
	Allele frequency	C vs. T	63/315	57/343	0.870	0.351	1.204 (0.815-1.777)
	Dominant model	CC+CT vs. TT	57/132	55/145	0.458	0.499	1.165 (0.748-1.815)
	Recessive model	CC vs. TT+CT	6/183	2/198	1.742	0.187	2.977 (0.589-15.036)

	Homozygous model	CC vs. TT	6/132	2/145	1.802	0.179	3.041 (0.600-15.426)
	Heterzygous model	CT vs. TT	51/132	53/145	0.132	0.716	1.088 (0.690-1.716)

PTOM: Posttraumatic osteomyelitis; OR: odds ratio, CI: confidence interval; NA: not available.
